# Effects of short-term ingestion of Russian Tarragon prior to creatine monohydrate supplementation on whole body and muscle creatine retention and anaerobic sprint capacity: a preliminary investigation

**DOI:** 10.1186/1550-2783-11-6

**Published:** 2014-02-26

**Authors:** Jonathan M Oliver, Andrew R Jagim, Ivo Pischel, Ralf Jäger, Martin Purpura, Adam Sanchez, James Fluckey, Steven Riechman, Michael Greenwood, Katherine Kelly, Cynthia Meininger, Christopher Rasmussen, Richard B Kreider

**Affiliations:** 1 Kinesiology Department, Texas Christian University, Fort Worth, TX 76129, USA; 2 Sport & Exercise Science Department, Gannon University, Erie, PA 16541, USA; 3 Phytolab GmbH & Co. KG, Vestenbergsgreuth, Germany; 4 Increnovo LLC, 2138 E Lafayette Pl, Milwaukee, WI 53202, USA; 5 Department of Health and Kinesiology, Exercise and Sport Nutrition Lab, Texas A&M University, College Station, TX 77843-4243, USA; 6 Department of Health and Kinesiology, Muscle Biology Laboratory, Texas A&M University, College Station, TX 77843-4243, USA; 7 Department of Health and Kinesiology, Human Countermeasures Laboratory, Texas A&M University, College Station, TX 77843-4243, USA; 8 Department of Systems Biology and Translational Medicine, Texas A&M Health Science Center, College Station, TX 77843-1114, USA

**Keywords:** Creatine supplementation, Russian Tarragon, Anaerobic capacity, Muscle creatine

## Abstract

**Background:**

Extracts of Russian Tarragon (RT) have been reported to produce anti-hyperglycemic effects and influence plasma creatine (Cr) levels while supplementing with creatine monohydrate (CrM). The purpose of this preliminary study was to determine if short-term, low-dose aqueous RT extract ingestion prior to CrM supplementation influences whole body Cr retention, muscle Cr or measures of anaerobic sprint performance.

**Methods:**

In a double-blind, randomized, and crossover manner; 10 recreationally trained males (20 ± 2 yrs; 179 ± 9 cm; 91.3 ± 34 kg) ingested 500 mg of aqueous RT extract (*Finzelberg, Andernach, Germany*) or 500 mg placebo 30-minutes prior to ingesting 5 g of CrM (*Creapure®, AlzChem AG, Germany*) twice per day for 5-days then repeated after a 6-week wash-out period. Urine was collected at baseline and during each of the 5-days of supplementation to determine urine Cr content. Whole body Cr retention was estimated from urine samples. Muscle biopsies were obtained for determination of muscle free Cr content. Participants also performed two 30-second Wingate anaerobic capacity tests prior to and following supplementation for determination of peak power (PP), mean power (MP), and total work (TW). Data were analysed by repeated measures MANOVA.

**Results:**

Whole body daily Cr retention increased in both groups following supplementation (0.0 ± 0.0; 8.2 ± 1.4, 6.5 ± 2.4, 5.6 ± 3.2, 6.1 ± 2.6, 4.8 ± 3.2 g · d^-1^; p = 0.001) with no differences observed between groups (p = 0.59). After 3 and 5-days of supplementation, respectively, both supplementation protocols demonstrated a significant increase in muscle free Cr content from baseline (4.8 ± 16.7, 15.5 ± 23.6 mmol · kg^-1^ DW, p = 0.01) with no significant differences observed between groups (p = 0.34). Absolute change in MP (9 ± 57, 35 ± 57 W; p = 0.031), percent change in MP (2.5 ± 10.5, 6.7 ± 10.4%; p = 0.026), absolute change in TW (275 ± 1,700, 1,031 ± 1,721 J; p = 0.032), and percent change in TW (2.5 ± 10.5, 6.6 ± 10.4%; p = 0.027) increased over time in both groups with no differences observed between groups.

**Conclusions:**

Short-term CrM supplementation (10 g · d^-1^ for 5-days) significantly increased whole body Cr retention and muscle free Cr content. However, ingesting 500 mg of RT 30-min prior to CrM supplementation did not affect whole body Cr retention, muscle free Cr content, or anaerobic sprint capacity in comparison to ingesting CrM with a placebo.

## Introduction

Research has consistently shown that creatine (Cr) supplementation is an effective strategy to increase muscle Cr content by up to 10-40% [1-3] which can significantly improve anaerobic performance, increase training volume, and enhance training adaptations [4-9]. By following a typical loading dose of 5 g of Cr, 4 times per day (total 20 g daily); muscle Cr content can significantly increase in as little as 3 to 7 days [2]. It has been suggested that the uptake of Cr into muscle is heavily influenced by initial intramuscular Cr concentrations and the type as well as amount of Cr ingested [10]. In this regard, studies have suggested that individuals who start Cr supplementation with low muscle Cr and phosphocreatine (PCr) content are more responsive to Cr supplementation. However, there are other factors that may influence the extent to which Cr is transported into the muscle cells, such as concentrations of glucose and insulin.

The most common form of Cr found in dietary supplements, food products, and referred to in scientific literature is creatine monohydrate (CrM) [10]. Despite consistent findings demonstrating CrM to be effective at increasing muscle Cr content [1-3,10], manufacturers have sought to develop alternative forms to increase market share. Several researchers have investigated the effect of these various forms of Cr in terms of Cr retention, uptake into the muscle cell, and effects on performance [11-15], confirming CrM as the most effective formulation [10]. (For review of alternative forms see [10]) Previous research has also shown that the addition of certain nutrients to Cr may improve Cr retention [16-19]. For example, researchers have found that the co-ingestion of 5 g of CrM with 93 g of glucose significantly increased Cr retention by 60% compared to CrM alone after 5 days of 20 g · d^-1^[17]. Similarly the addition of certain macronutrients have also been shown to improve Cr retention [18]. Steenge et al. [18] found that the addition of 96 g of carbohydrates and/or 47 g of carbohydrates with 50 g of protein to 20 g of CrM daily improved Cr retention by roughly 25% (p < 0.05) compared to 5 g carbohydrates. Results of the study suggest that higher insulin levels, in response to the additional macronutrients, may augment Cr uptake into the muscle.

While co-ingesting large amounts of carbohydrate and/or protein with Cr has been reported to augment muscle and/or whole body Cr retention, some athletes or recreationally active individuals may be interested in lower-calorie strategies to improve Cr uptake. Recently there has been an interest in the effects of combining Cr with additional ingredients to improve Cr uptake and retention. For example, Greenwood and associates [16] found that the co-ingestion of 1 g of D-pinitol (a plant extract with insulin-like properties) per day with CrM (20 g/d) for 3 days significantly improved Cr absorption and retention compared to CrM alone and a placebo. Ethanolic or aqueous extracts of Russian Tarragon (RT) (*artemisia dracunculus*) have been purported to have anti-hyperglycemic effects. Theoretically, co-ingestion of RT with Cr may help augment Cr uptake [20,21]. To support this theory, Jäger et al. [20] found that plasma Cr levels were reduced when RT was combined with CrM compared to CrM alone, suggesting an increase in Cr uptake. Therefore, the purpose of this study was to examine whether a low dose aqueous RT extract ingested 30 minutes prior to CrM intake during a 5-day loading phase significantly affected whole body Cr retention and/or anaerobic capacity in healthy, recreationally active males when compared to CrM ingestion alone.

## Methods

### Experimental design

The study was conducted in a double-blind, randomized, and crossover manner. The independent variable was RT extract supplementation. Dependent variables included intramuscular Cr concentration, whole body Cr retention, and anaerobic sprint performance capacity. Participants who qualified for the study participated in a familiarization session in which the study was explained following written consent. Participants were then randomly assigned to ingest 2 × 500 g of a placebo (P) or RT, 30-minutes prior to ingesting 5 g of CrM, twice a day for a 5-day period. Urine was collected at baseline and during each of the 5-days of supplementation to determine urine Cr content. At baseline and on days 3 and 5 of supplementation, participants returned to the lab for a muscle biopsy and a Wingate anaerobic capacity test. Urine was collected daily throughout the supplementation period for determination of whole-body Cr retention. Dietary intake was not controlled for but participants were asked to maintain normal dietary practices and record all food intake four days prior to the study and then replicate these practices prior to the next testing session. It has been reported that Cr stores return to baseline after approximately 30 days following cessation of supplementation [1]. To ensure a return to baseline levels, participants then completed a 6-week wash-out period prior to repeating the experiment following the alternate supplementation regimen.

### Participants

**Figure 1:**
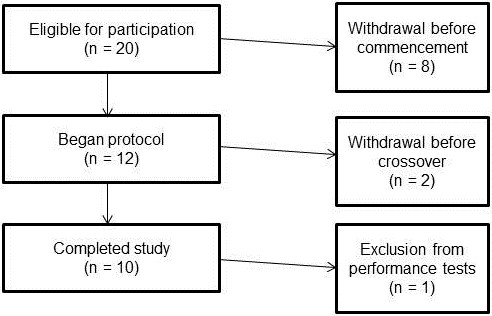
Consort diagram.

A consort diagram is provided in Figure 1 outlining reasons for drop out and/or exclusion. Reasons for drop out included scheduling conflicts with no one reporting drop out due to supplementation protocol. Ten apparently healthy recreationally trained males (20 ± 2 yrs; 179 ± 9 cm; 91.3 ± 34 kg) with no self-reported recent history of Cr supplementation completed the entire study. Participants were not allowed to participate in this study if they had any metabolic disorder including known electrolyte abnormalities; heart disease, arrhythmias, diabetes, thyroid disease, or hypogonadism; a history of hypertension, hepatorenal, musculoskeletal, autoimmune, or neurologic disease; if they were taking thyroid, anti-hyperlipidemic, hypoglycemic, anti-hypertensive, anti-inflammatory, or androgenic medications; or, if they had taken dietary supplements containing creatine within three months prior to the start of the study. Participants were recruited from the student population and from area fitness facilities. All participants responding to advertisement and met eligibility criteria were informed of the requirements of the study and invited to a familiarization session. Following explanation of the study procedures, participants signed an informed consent statement in compliance with the Human Subjects Guidelines of Texas A&M University and the American College of Sports Medicine. Participants then completed demographic, health history, and exercise history forms followed by performance of the Wingate anaerobic capacity test (WAnT), which served as familiarization for testing sessions. None of the participants reported training for a sport and/or recreational event during the time of the study. Participants were asked to maintain their normal recreational activities throughout the duration of the study.

### Testing sessions

Following familiarization, participants were provided urine collection containers in order to collect 24 hour urine samples throughout the study. Participants reported daily to the laboratory to drop off urine samples and turn in their reported supplement side effects form as well as the requested supplement adherence questionnaire. Muscle biopsies and exercise testing occurred on days 0, 3, and 5. Participants were instructed to refrain from exercise for 48 hours and fast for 8-hours prior to testing sessions. Muscle biopsies were obtained on day 0, 3, and 5. Since this was a cross-over design, the same number of biopsies were obtained on the contralateral leg after the washout period; totaling 6 biopsies per participant. Following muscle biopsy procedures, participants performed two 30-second WAnT separated by 3 minutes.

### Supplementation protocol

Participants were randomly assigned to ingest, in a double-blind and cross-over manner, capsules containing 500 mg of an aqueous extract of RT (*Finzelberg, Andernach, Germany*) or a placebo (P) (Luvos Heilerde) with CrM [*Creapure, AlzChem, Trostberg, Germany*]. The RT and P supplements were provided in capsules and two (2x) were consumed 30-minutes prior to ingesting 5 grams of CrM two times daily for 5 days. After a 6-week wash out period, participants repeated the experiment and consumed the alternate supplement capsules prior to CrM supplementation. Participants were instructed to ingest the supplements at 0800 and 2000 each day in order to standardize supplement intake/absorption for the 5 day period. Supplements were comprised of similar texture, taste, and appearance and placed in generic single serving packets for double-blind administration. The supplements were prepared for distribution by the supporting sponsors of this research endeavor. Supplementation compliance was monitored by having the participants return empty containers of the supplement at the end of each testing session. In addition, participant compliance was verified by collecting daily supplement adherence questionnaires when dropping off urine containers. Participants were then provided the supplement dosage for the next day.

### Procedures

#### Muscle and urine samples

Following familiarization, participants were provided eight, 3 L urine collection containers in order to collect 24 hour urine samples for baseline (day 0) and day 1, 2, 3, 4, and 5. Participants were also requested to record the number of times they urinated each day. The 24 hour baseline urine sample time parameter was initiated at 0800 am the day before the supplementation protocol began. Participants were asked to refrigerate their urine samples during the 24 hour time period. Participants reported daily to the laboratory between 0700 and 0800 to drop off urine samples. Whole body creatine retention was estimated as the difference between orally ingested CrM (10 g · d^-1^) and the amount of Cr excreted daily in urine as previously described [22].

Muscle biopsies were obtained using a modified Bergstrom needle biopsy technique following standard procedures [23]. Percutaneous muscle biopsies (50–70 mg) were obtained from the middle portion of the vastus lateralis muscle at the midpoint between the patella and the greater trochanter of the femur at a depth between 1 and 2 cm into the muscle. For the remaining two biopsies, attempts were made to extract tissue from approximately the same location as the initial biopsy by using the pre-biopsy scar, depth markings on the needle, and successive incisions that were made approximately 2 cm proximal to the former site. The initial leg was chosen by the flip of a coin and the contralateral leg was used during the cross-over. After removal of adipose tissue, the muscle specimens were immediately frozen in liquid nitrogen and then stored at–80°C for later analysis. Three muscle samples were obtained (Days 0, 3, & 5) with the same number repeated during crossover on the contralateral leg for a total of six muscle biopsies.

Muscle tissue samples were prepared for spectrophotometric analysis for Cr using methods developed by Harris and colleagues [22,24,25]. Briefly, approximately 50–70 mg of muscle tissue was cut and transferred into a microfuge tube, followed by a dehydration process in a vacuum centrifuge (*Savant ISS110 SpeedVac Concentrator, Thermo Scientific, Milford, MA*) and centrifuged for 18–24 hours. Connective tissue was removed from the dried samples which were then grinded into a powder in a porcelain plate and placed into pre-weighed microfuge tubes. Muscle metabolites were extracted in a 0.5 M perchloric acid/ 1 mM EDTA solution on ice for 15 minutes, while periodically vortexing. Samples were then centrifuged at 7,000 rpm for 5 minutes. The supernatant was transferred into a pre-weighed microfuge tube and neutralized with 2.1 M KHCO3/0.3 M MOPS solution. The samples were then centrifuged again at 7,000 rpm for 5 minutes and the supernatant was removed and placed into microfuge tubes and frozen at–80°C.

Muscle extracts and urine samples were assayed for Cr in the presence of 50 mM imidazole buffer, pH 7.4; 5 mM magnesium chloride; 20 mM potassium chloride; 25 μM phosphoenolpyruvate; 200 μM ATP; 45 μM NADH; 1250 U/mL lactate dehydrogenase; 2000 U/mL pyruvate kinase. The assay was carried out in a standard fluorescence microplate reader using 10 μL of sample to 1 mL of reagent. The reactant solution was vortexed and read using a fluorometer (*Shimadzu RFMini 150, Japan*) with an excitation wavelength of 340 nm and an emission wavelength of 460 nm for baseline absorbance values. Five μL of CK (25 μ/mg) was added to 1 mL of the above buffer and stabilized using 1 mL of reagent. After 10 minutes the plate was read again for post-reaction absorbance values. Test to test reliability of duplicate muscle Cr assays was 0.01 ± 0.10 (r = 0.81) with a coefficient of variation of 2.62. Test to test reliability of duplicate of urine Cr assays was 0.01 ± 0.04 (r = 0.99) with a coefficient of variation of 1.13. We also assayed muscle and urine samples for PCr, but several values were out of normal ranges in both muscle and urine. Further, there was large variability in the values observed. This suggested lack of validity of this assay and therefore, these data were not reported.

### Performance tests

Participants performed a 30-second Wingate anaerobic capacity sprint test on a Lode Excalibur Sport 925900 cycle ergometer (*Lode BV, Groningen, The Netherlands*) at a standardized work rate of 7.5 J/kg/rev. The seat position was recorded for each participant and used in all subsequent performance tests. Each participant was asked to pedal as fast as possible prior to application of the workload and sprint at all-out maximal capacity during the 30-second test. Test-to-test variability in performing repeated Wingate anaerobic capacity tests in our laboratory yielded correlation coefficients of *r =* 0.98 ± 15% for mean power [12]. Participants practiced the anaerobic capacity test during the familiarization session to minimize learning effects. One participant opted out of performance testing due to a prior injury not resulting from participation in the study.

### Side effect assessment

Participants were given daily questionnaires on how well they tolerated the supplement, how well they followed the supplement protocol, and if they experienced any medical problems/symptoms during the study. Compliance to the supplementation protocol was monitored daily as participants returned to the lab to hand in urine jugs and complete a daily questionnaire. After completing the compliance procedures, participants were given the required supplements and dosages for the following supplementation period.

### Statistical analysis

All statistical analysis was performed using SPSS V.20 (*Chicago, IL*) software. Study data were analyzed by Multivariate Analysis of Variance (MANOVA) with repeated measures. Overall MANOVA effects were examined using the Wilks’ Lambda time and group x time p-levels as well as MANOVA univariate ANOVA group effects. Greenhouse-Geisser univariate tests of within-subjects time and group × time effects and between-subjects univariate group effects were reported for each variable analyzed within the MANOVA model. The sum of daily-whole body Cr retention during the study was evaluated by a studentized t-test to determine any differences between groups. Data were considered statistically significant when the probability of type I error was 0.05 or less. If a significant group, treatment, and/or interaction alpha level was observed, Tukey’s least significant differences (LSD) post-hoc analyses was performed to determine where significance was obtained.

## Results

### Urinary creatine excretion and retention

Table 1 presents daily urinary Cr excretion and whole-body Cr retention data. A significant time effect was observed in both daily urinary Cr excretion (p = 0.001) and whole-body retention (p = 0.001), in which post hoc analysis demonstrated similar time effects throughout the supplementation protocol (Table 1). No significant differences were observed between groups (p = 0.82) in total whole-body Cr retention throughout the supplementation protocol, 31.7 ± 11.1 and 30.6 ± 9.9 grams for P + CrM and RT + CrM, respectively.

**Table 1:** Daily urinary creatine (Cr) excretion and retention

		**Day**							
**Variable**	**Group**	**0**	**1**	**2**	**3**	**4**	**5**		**p-level**
Urinary Cr Excreted (g∙day^-1^)	P + CrM	0.3 ± 0.4	1.9 ± 1.60	3.5 ± 2.300	4.7 ± 3.3000	3.2 ± 2.800	5.0 ± 3.4000	Time	0.001
	RT + CrM	0.5 ± 0.6	1.7 ± 1.10	3.4 ± 2.700	4.2 ± 3.3000	4.6 ± 2.200	5.4 ± 3.2000	Group	0.801
	Combined	0.4 ± 0.5	1.8 ± 1.4*	3.5 ± 2.4*†	4.4 ± 3.2*†‡	3.9 ± 2.6*†	5.2 ± 3.2*†‡	GxT	0.59
Whole body Cr Retention (g∙day^-1^)	P + CrM	0.0 ± 0.0	8.1 ± 1.60	6.5 ± 2.300	5.3 ± 3.3000	6.8 ± 2.800	5.0 ± 3.4000	Time	0.001
	RT + CrM	0.0 ± 0.0	8.3 ± 1.10	6.6 ± 2.700	5.8 ± 3.3000	5.4 ± 2.200	4.6 ± 3.2000	Group	0.82
	Combined	0.0 ± 0.0	8.2 ± 1.4*	6.5 ± 2.4*†	5.6 ± 3.2*†‡	6.1 ± 2.6*†	4.8 ± 3.2*†‡	GxT	0.59

(n = 10).

Values are means ± standard deviations. (n = 10) Greenhouse-Geisser time and group x time (G x T) interaction p-levels are reported with univariate group p-levels. *Significantly different than Day 0. †Significantly different than Day 1. ‡Significantly different than Day 2.

### Muscle creatine analysis

Table 2 presents muscle free Cr content data. Sufficient muscle samples were obtained to measure baseline and subsequent creatine on all (n = 10) participants. A MANOVA was run on muscle Cr expressed in mmol · kg^-1^ DW, changes from baseline expressed in mmol · kg^-1^ DW and percent changes from baseline. An overall MANOVA time effect (Wilks’ Lambda p = 0.03) was observed with no significant overall group × time interactions (Wilks’ Lambda p = 0.34). MANOVA univariate analysis revealed significant time effects in muscle free Cr content expressed in absolute terms (p = 0.019), changes from baseline (p = 0.019), and percent changes from baseline (p = 0.006), in which post hoc analysis revealed a significant increase in muscle free Cr content by day 5. No significant differences were observed between groups.

**Table 2:** Muscle free creatine (Cr) levels

**Variable**	**Group**	**0**	**Day 3**	**5**		**p-level**
**Cr** (mmol∙kg^-1^ DW)	P + CrM	72.1 ± 26.0	81.2 ± 26.0	94.9 ± 40.5	Time	0.019
	RT + CrM	103.0 ± 21.1	103.2 ± 27.2	111.0 ± 19.0	Group	0.049
	Combined	87.5 ± 28.0	92.3 ± 28.2	102.9 ± 31.9*	GxT	0.34
**Cr** (Δ mmol∙kg^-1^ DW)	P + CrM	0.0 ± 0.0	9.3 ± 14.3	22.8 ± 28.2	Time	0.019
	RT + CrM	0.0 ± 0.0	0.3 ± 18.4	8.1 ± 16.2	Group	0.097
		0.0 ± 0.0	4.8 ± 16.7	15.5 ± 23.6*	GxT	0.34
**Cr** (Δ%)	P + CrM	0.0 ± 0.0	21.1 ± 30.0	37.3 ± 41.7	Time	0.008
	RT + CrM	0.0 ± 0.0	0.7 ± 20.5	9.6 ± 18.1	Group	0.035
	Combined	0.0 ± 0.0	10.9 ± 27.1	23.5 ± 34.4*	GxT	0.13

(n = 10).

Values are means ± standard deviations. (n = 10) Δ represents change from baseline values. Data were analyzed by MANOVA with repeated measures. Greenhouse-Geisser time and group x time (G x T) interaction p-levels are reported with univariate group p-levels. *Significantly different than Day 0.

### Anaerobic capacity

Table 3 presents changes in peak power, mean power, and total work as observed during the 30-second WAnT. One participant did not participate in performance tests during crossover and thus all data were excluded for analysis (n = 9). No overall time or time x group effects were observed for peak power (Wilks’ Lambda p = 0.40 and p = 0.52, respectively). An overall MANOVA time effect (Wilks’ Lambda p = 0.025 and p = 0.025) was observed for mean power and total work, respectively, with no overall group x time interactions observed. MANOVA univariate analysis revealed significant time effects in mean power and total work. Post hoc analysis revealed significant increases in both mean power and total work by day 5. No significant differences were observed between groups.

**Table 3:** Changes in peak power, mean power, and total work during Wingate

**Variable**	**Group**	**0**	**Day 3**	**5**		**p-level**
**Peak power** (W)	P + CrM	1,472 ± 451	1,435 ± 182	1,380 ± 244	Time	0.68
	RT + CrM	1,559 ± 213	1,565 ± 398	1,519 ± 339	Group	0.31
	Combined	1,515 ± 345	1,500 ± 307	1,450 ± 295	GxT	0.92
**Mean power** (W)	P + CrM	591 ± 94	599 ± 89	642 ± 8300	Time	0.031
	RT + CrM	590 ± 103	601 ± 78	608 ± 9600	Group	0.79
	Combined	591 ± 96	600 ± 81	625 ± 89*†	GxT	0.27
**Total work** (J)	P + CrM	17,742 ± 2,822	17,970 ± 2,663	19,264 ± 2,48200	Time	0.032
	RT + CrM	17,706 ± 3,098	18,029 ± 2,339	18,246 ± 2,88800	Group	0.79
	Combined	17,724 ± 2,875	17,999 ± 2,432	18,755 ± 2,664*†	GxT	0.27

(n = 9).

Values are means ± standard deviations. Δ represents change from baseline values. Data were analyzed by MANOVA with repeated measures. Greenhouse-Geisser time and group x time (G x T) interaction p-levels are reported with univariate group p-levels. *Significantly different than Day 0. †Significantly different than Day 3.

### Side effect assessment

For all participants who completed the study, supplement compliance was 100%. No side effects were reported for the duration of the study.

## Discussion

Ethanolic and aqueous extracts of Russian Tarragon (RT) (*artemisia dracunculus*) have been purported to have anti-hyperglycemic effects [21,26,27]. A previous study found that ingesting this same dose of RT with CrM resulted in a greater reduction in plasma Cr levels suggesting greater uptake [20]. The purpose of this study was to examine whether a low dose aqueous RT extract ingested 30 minutes prior to CrM intake during a 5-day loading phase significantly affected whole body Cr retention and/or anaerobic capacity in healthy, recreationally active males when compared to CrM ingestion alone. Our preliminary findings indicate that ingesting 500 mg RT 30-min prior to CrM supplementation did not affect whole body Cr retention or muscle free Cr content during a short-period of CrM supplementation (10 g · d^-1^ for 5-days) in comparison to ingesting a placebo prior to CrM supplementation. Further, results of this preliminary study indicate that ingesting 500 mg RT 30-min prior to CrM supplementation had no additive effects on anaerobic sprint capacity in comparison to ingesting CrM with a placebo.

While oral ingestion of CrM alone increases total muscle Cr content, the addition of other nutrients has been shown to enhance the absorption, thereby increasing muscle Cr content to a greater degree than CrM alone. Green et al. [17] demonstrated that ingesting 5 g CrM followed by 93 g simple carbohydrate (glucose and simple sugars) resulted in an increase in muscle Cr content compared to CrM alone. Later investigations have shown that a lesser amount of carbohydrate (35 g) with each dose of CrM may promote greater adaptations than CrM alone. Based on these findings, it has been hypothesized that Cr retention during supplementation may be mediated in part by the insulin pathway. In support of this hypothesis, Steenge et al. [28] demonstrated that insulin can enhance muscle Cr accumulation, but only when present at physiologically high or supraphysiological concentrations. While co-ingesting large amounts of carbohydrate and/or protein with Cr have been reported to promote muscle Cr retention, some athletes or recreationally active individuals may be interested in lower-calorie strategies to improve Cr uptake. Greenwood et al. [16] found that the co-ingestion of 1 g of D-Pinitol (a plant extract with insulin-like properties) per day with CrM (20 g/d) for 3 days significantly improved whole body Cr retention. While D-Pinitol provides a non-caloric substitute to other higher calorie nutrients, it is relatively expensive. Further, no other studies have demonstrated D-Pinitol to increase total muscle Cr.

Extracts of RT have been purported to have anti-hyperglycemic effects. The effect of RT on carbohydrate metabolism is most noted in animal models. For instance, Ribnicky et al. [27] showed the ethanolic extract of RT to reduce insulin concentrations by 33% compared to 48% and 52% for the antidiabetic drugs troglitzaone and metformin, respectively. Further, this same research group has shown ethanolic RT to significantly lower blood glucose concentrations by 20% in streptozotocin-induced diabetic mice, compared to control. However, the dosage in that study was significantly greater than the present study (500 mg/kg bodyweight). Further evidence of the anti-hyperglycemic effects of RT has been provided by Pischel et al. [29]. Using the same aqueous extract of RT and dosage used in the current study, Pischel et al. [29] reported lower blood glucose levels in both animals and humans (albeit non-statistically) following ingestion.

While the antidiabetic properties of RT are a relatively new discovery [30], current investigations are focusing on alterations in the insulin pathway. Given the purported role of insulin in enhanced muscle Cr accumulation, RT may serve as a means to augment Cr retention without the ingestion of carbohydrate and the resulting greater caloric intake. Jäger et al. [20] demonstrated a significant reduction in plasma Cr levels following ingestion of similar dose of RT followed by CrM compared with CrM alone. However, the current study suggests no additive effect of RT when combined with CrM on muscle Cr retention. Despite similar RT and CrM dosing strategy, 10 g · day^-1^ in current study compared to 60 g · kg bodyweight (just over 10 g using current participant average weight), measuring muscle Cr content demonstrated no additive effect of RT. It must be noted that despite there not being a statistically significant difference, the baseline muscle free Cr during the supplementation of RT and CrM appears to be slightly higher than CrM alone. There is no doubt large inter-individual differences in the change in Cr in muscle as evidenced by the work of Harris et al. [31] and Greenhaff et al. [2]. More importantly, Greenhaff et al. [2] demonstrated that any measureable effect on PCr resynthesis as a result of Cr ingestion was only observed in individuals demonstrating greater than a 20 mmol•kg^-1^ increase in TCr. Thus, the apparent higher baseline free Cr may have contributed to the current findings.

Despite finding no additive effect of RT, a novel aspect of the current study was the finding that ingesting as little as 5 g of CrM twice daily (i.e., 10 g · d^-1^) increased total muscle Cr content by 23.5 ± 34.5%. This dosing strategy was based on the previous study by Jäger et al. [20]. To the authors’ knowledge, this is the first study to report significant increases in muscle Cr following low dose supplementation. This occurred despite being lower than dosage strategies used in previous studies (20 g · d^-1^ or 0.3 g · kg^-1^ · bw^-1^) [5,9]. Harris et al. [31] were the first to demonstrate supplementation with 5 g CrM taken orally 4–6 times per day for two or more days resulted in a significant increase in muscle Cr content. The authors further noted the greatest change occurred in those individuals with low initial total Cr content. The increase in muscle Cr content observed in the current study is similar to values reported in the literature with higher loading doses (25 ± 3%) [2]. Further, we observed a significant improvement in both MP and TW by 2-7% following a lower dosing strategy suggesting that this level of Cr supplementation may be sufficient to affect anaerobic exercise capacity. This finding furthers the research in the area of the optimal loading phase dosing strategy to effectively increase muscle Cr stores.

In summary, the most important finding in this study were as little as 5 g CrM taken twice daily for 3–5 days increases total muscle Cr, whole body Cr retention, and improves MP and TW. However, results of this pilot study do not support contentions that ingesting 500 mg of RT prior to CrM supplementation enhances whole body Cr retention, muscle free Cr content, or provides an additive effect on anaerobic sprint capacity during a short-period of CrM supplementation. Additional research is needed with a larger sample size to examine: 1.) whether ingestion of greater amounts of RT prior to and/or in conjunction with CrM ingestion would affect Cr retention; 2.) whether ingestion of RT with CrM over longer periods of time would affect Cr retention; and, 3.) whether co-ingesting RT with CrM and carbohydrate may reduce the need for ingesting large amounts of carbohydrate with CrM in order to promote greater Cr retention.

## Competing interests

Martin Bauer Group, Finzelberg GmbH & Co. KG. provided funding for this study through a research grant to Texas A&M University. All researchers involved independently collected, analyzed, and interpreted the results from this study and have no financial interests concerning the outcome of this investigation. RBK has received grants as Principal Investigator through institutions with which he has been affiliated to conduct exercise and nutrition related research, has served as a legal and scientific consultant, and currently serves as a scientific consultant for Woodbolt International (*Bryan, TX*). MP, IP, and RJ have been named as inventors on pending patents by the Martin Bauer Group. Remaining co-authors have no competing interests to declare. Data from this study have been presented at the International Society of Sports Nutrition Annual meeting and have not been submitted for publication to any other journals. Publication of these findings should not be viewed as endorsement by the investigators or their institutions of the nutrients investigated.

## Authors’ contributions

JMO served as the study coordinator, oversaw all testing, and assisted in data analysis and writing of the manuscript. ARJ assisted in data collection and statistical analysis. IP, RJ, and MP assisted in the experimental design, data analysis, and manuscript preparation. AS assisted with data collection JF and SR supervised the biopsy procedures. MG assisted in experimental design, data analysis, and manuscript preparation. KK supervised muscle assays and CM served as a collaborating scientist. CR served as lab coordinator and oversaw data collection and quality control of the study. RBK served as Principal Investigator and contributed to the design of the study, statistical analysis, manuscript preparation, and procurement of external funding. All authors read and approved the final manuscript.
